# Efficient Expression of HIV in Immunocompetent Mouse Brain Reveals a Novel Nonneurotoxic Viral Function in Hippocampal Synaptodendritic Injury and Memory Impairment

**DOI:** 10.1128/mBio.00591-19

**Published:** 2019-07-02

**Authors:** Jennifer Kelschenbach, Hongxia He, Boe-Hyun Kim, Alejandra Borjabad, Chao-Jiang Gu, Wei Chao, Meilan Do, Leroy R. Sharer, Hong Zhang, Ottavio Arancio, Mary Jane Potash, David J. Volsky

**Affiliations:** a Department of Medicine, Infectious Diseases Division, Icahn School of Medicine at Mount Sinai, New York, New York, USA; b Department of Pathology and Laboratory Medicine, Rutgers New Jersey Medical School, Newark, New Jersey, USA; c Department of Pathology and Cell Biology, Columbia University Medical Center, New York, New York, USA; Department of Biochemistry and Molecular Biology, Miller School of Medicine, University of Miami; Albert Einstein College of Medicine

**Keywords:** EcoHIV, HAND, mouse models, radial arm water maze, synaptodendritic injury

## Abstract

HIV causes neurodegeneration and dementia in AIDS patients, but its function in milder cognitive impairments in virologically suppressed patients on antiretroviral therapy is unknown. Such patients are immunocompetent, have low peripheral and brain HIV burdens, and show minimal brain neuropathology. Using the model of HIV-related memory impairment in EcoHIV-infected conventional mice, we investigated the neurobiological and cognitive consequences of efficient EcoHIV expression in the mouse brain after intracerebral infection. HIV integrated and persisted in an expressed state in brain tissue, was detectable in brain monocytic cells, and caused neuroinflammatory responses and lasting spatial, working, and associative memory impairment. Systemic antiretroviral treatment prevented direct brain infection and memory dysfunction indicating the requirement for HIV expression in the brain for disease. Similarly inoculated murine leukemia virus used as a control replicated in mouse brain but not in monocytic cells and was cognitively benign, linking the disease to HIV-specific functions. Memory impairment correlated in real time with hippocampal dysfunction shown by defective long-term potentiation in hippocampal slices *ex vivo* and with diffuse synaptodendritic injury in the hippocampus reflected in significant reduction in microtubule-associated protein 2 and synapsin II staining. In contrast, there was no evidence of overt neuronal loss in this region as determined by neuron-specific nuclear protein quantification, TUNEL assay, and histological observations. Our results reveal a novel capacity of HIV to induce neuronal dysfunction and memory impairment independent of neurotoxicity, distinct from the neurotoxicity of HIV infection in dementia.

## Abstract (precis)

HIV neuropathogenesis has been attributed in large measure to neurotoxicity of viral proteins and inflammatory factors produced by infected monocytic cells in the brain. We show here that HIV expression in mouse brain causes lasting memory impairment by a mechanism involving injury to hippocampal synaptodendritic arbors and neuronal function but not overt neuronal loss in the region. Our results mirror the observation of minimal neurodegeneration in cognitively impaired HIV patients on antiretroviral therapy and demonstrate that HIV is nonneurotoxic in certain brain abnormalities that it causes. If neurons comprising the cognition-related networks survive HIV insult, at least for some time, there is a window of opportunity for disease treatment.

## INTRODUCTION

HIV infection causes brain abnormalities known as HIV-associated neurocognitive disorders (HAND) that include HIV-associated dementia (HAD) and two non-HAD neurocognitive impairments (NCI), asymptomatic neurocognitive impairment (ANI) and mild neurocognitive disorder (MND) (1). Individuals with stable virologic suppression by antiretroviral therapy (ART) rarely progress to HAD, but about 50% of them will develop NCI (2–4). ANI, diagnosed only in neuropsychological tests, is the largest NCI category in patients on ART, and the number of cases is also increasing (5, 6). ANI poses an increased risk of symptomatic disease (7, 8), and aging increases the severity of both disorders (3, 9, 10), indicating substantial long-term health consequences.

The frequency of NCI raises the question of how HIV causes this impairment. Studies in HAD patients and animal models of HIV encephalitis (HIVE) linked dementia to HIV replication, CD4^+^ T cell depletion/immunodeficiency, and neuroinflammatory and neurotoxic effects of HIV proteins produced by infected cells in the brain (reviewed in references 11 and 12). Although NCI was described in AIDS patients independent of dementia (13, 14), the failure of ART to prevent NCI (2, 3) suggests routes of HIV neuropathogenesis unlinked to virus burden and immunodeficiency and different from HAD/HIVE (15, 16). Further mechanistic differences are apparent in the limited neurodegeneration in NCI patients on ART (15, 17) and are reflected in the stable cognitive disease course in the majority of individuals on ART monitored for 3 to 4 years (3, 9, 10). Brain pathology studies on NCI revealed that NCI correlates better with cortical synaptodendritic simplification than with prominent neuronal loss (13, 18, 19). Neuronal dysfunction independent of neuronal loss is now considered the major pathobiological feature distinguishing NCI from HAD (6). Collectively, these findings suggest that HIV may act differently in causing NCI and HAD. Given that ART suppresses HIV replication, part of this difference could be quantitative, but the demonstration of mild NCI in AIDS patients with high virus burdens (13, 18) speaks against it. In addition, HIV RNA can be detected in brain tissues and CSF from NCI patients with undetectable HIV in plasma (3, 20), suggesting ongoing virus expression in the central nervous system. Alternatively, HIV could cause nonnecrotic neuronal dysfunction or neurodegeneration depending on host conditions, for example being related to aging (21).

We have employed chimeric HIV, EcoHIV (22), to investigate NCI in conventional immunocompetent mice. EcoHIV-infected mice mount protective antiviral immune responses and have low virus burdens and normal brain histopathology, yet they manifest learning and memory dysfunctions (named murine NCI) that resemble human NCI (23–25). As shown recently, murine NCI correlates with synaptodendritic injury in the hippocampus and both the injury and NCI can be reversed by intranasal insulin treatment, suggesting that some injured neurons remain viable (26). Here, we used intracerebral (i.c.) virus delivery for efficient EcoHIV infection in immunocompetent mouse brain (24, 27) to directly evaluate the neurobiological and cognitive effects of HIV expression in the mouse brain.

## RESULTS

### EcoHIV but not MLV impairs memory in wild-type mice.

**FIG 1:**
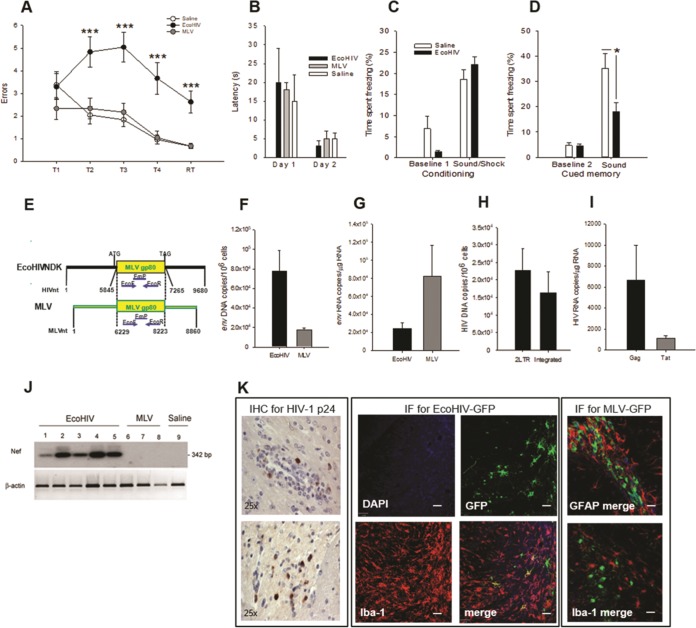
EcoHIV and MLV efficiently replicate in mouse brain but only EcoHIV causes learning and memory impairments. (A and B) The RAWM test of mice inoculated intracranially with EcoHIV (closed circles), MLV (gray circles), or saline (open circles). Testing was conducted over 12 days starting 10 days after infection (*n* = 8 per group). (A) Mean number of errors ± the standard errors of the mean (SEM) made on the last 3 days of RAWM testing with the hidden platform. T1 to T4 denote acquisition trials, and RT denotes the retention trial performed after a 30-min delay. F_(2,71)_ = 8.951, *P* < 0.001/T2, F_(2,71)_ = 14.171, *P* < 0.001/T3, F_(2,71)_ = 11.444, *P* < 0.001/T4, F_(2,71)_ = 14.671, *P* < 0.001/R; (***, *P* < 0.001). (B) Mean latencies ± the SEM in finding the visible platform on trial 4 on days 1 and 2 of visible platform testing. (C and D) The auditory-cued fear conditioning test 2 weeks after intracranial inoculation with EcoHIV or saline (*n* = 8 per group). The figure shows the mean percentages of time spent freezing ± the SEM during paired sound-shock conditioning sessions on day 1 of the test (C) and sound (cued) response sessions in a new context on day 2 of the test (D). (E) Schematic representation of the shared MLV gp80 *env* gene in EcoNDK and MLV. (F and G) Quantification of EcoHIV (black bars) and MLV (gray bars) showing DNA or RNA burdens, respectively, in the brain tissues of mice after completion of the RAWM test shown in panels A and B. The *env* DNA or RNA region was amplified by QPCR or RT-QPCR, respectively; results are mean copies ± the SEM. (H to J) In a parallel experiment, three mice each were infected intracranially with EcoHIV, and 2 weeks later the brain tissues were tested by QPCR for HIV 2LTR and integrated DNA (H), by RT-QPCR for HIV Gag and Tat RNA (I), and by Southern blot hybridization for Nef transcript (J). The results in panels H and I are mean copies ± the SEM; β-actin RNA amplified in parallel served as a loading control in panel J. (K) Detection and cellular localization of viral proteins in mouse brain sections 2 weeks after intracranial infection of three to four mice each with EcoHIV (left panels), EcoHIV-GFP (middle panels), or MLV-GFP (Right panels). Representative images from five to seven sections in each mouse are shown. (Left panel) Detection of HIV p24 by immunohistochemical (IHC) staining evaluated by light microscopy. Note the p24-positive cells near a perivascular lesion (top) and in the brain parenchyma (bottom). Magnification, ×25. (Middle panel) Localization of HIV-encoded GFP in monocytoid cells by costaining for macrophage marker Iba-1 (red), GFP (green), and nuclear marker DAPI (blue) and evaluation by fluorescence microscopy. Individual channels and a merge image are shown. (Right panel) Detection of MLV-encoded GFP by costaining for GFP (green), nuclear marker DAPI (blue), and either astrocyte marker GFAP (red, upper image) or macrophage marker Iba-1 (red, lower image) and evaluation by fluorescence microscopy. Representative merged images are shown. Note the absence of colocalization of GFP with either GFAP or Iba-1.

Animal lentivirus models of HIV brain disease generally reproduce HIVE in immunodeficient hosts with high virus brain burdens (28). To test the pathogenicity of brain-resident HIV in an immunocompetent host, wild-type 129X1 mice were inoculated intracerebrally with EcoHIV or murine leukemia virus (MLV) (24, 27) and, starting 10 days after infection, the animals were tested in a RAWM for visuospatial learning and memory (Fig. 1A and B). MLV, the donor of viral envelope in EcoHIV (22), was used both as an unrelated retrovirus control and to control for effects of MLV envelope in EcoHIV. Because the location of the submerged platform is altered randomly on each day of the test, the animals must use working memory to learn the platform location (29–31). Saline- and MLV-inoculated mice learned and retained acquired information equally well, but EcoHIV-infected mice were significantly impaired at all testing stages (significant main effect of trial F_(5,359)_ = 9.152, *P* < 0.001 and infection F_(2,359)_ = 40.041, *P* < 0.001) (Fig. 1A). The infected and control mice were equally adept in finding the visible platform (Fig. 1B), suggesting that neither EcoHIV nor MLV affected the visual, motor, or motivational circuits in the brain. To examine another cognitive domain, infected mice were tested in a fear conditioning (FC) test (Fig. 1C and D) for assessment of amygdala-dependent fear memory (32, 33). Control and infected mice learned equally well to associate electric shock and sound during the conditioning session on day 1 (Fig. 1C), but infected/conditioned animals spent less time “freezing” (motionless) than control/conditioned mice when exposed the next day to a sound cue alone (Fig. 1D; *P* = 0.026), suggesting the impairment of sound-cued associative memory in these mice. Thus, EcoHIV infection of mouse brain impairs hippocampus- and amygdala-dependent memory circuits, while MLV was cognitively benign.

### EcoHIV and MLV replicate well in mouse brain, but only EcoHIV infects macrophages/microglia.

We next inquired whether the difference between EcoHIV and MLV in cognitive disease outcomes could be explained by their infection phenotypes in mouse brain (Fig. 1E to K). Quantitative PCR (QPCR) amplification of a common region in the gp80 gene depicted in Fig. 1E allows equal detection of both viral genomes. Both EcoHIV- and MLV-infected mice had high *env* DNA (Fig. 1F) and RNA burdens (Fig. 1G) at the completion of the behavioral experiment shown in Fig. 1A and B, demonstrating efficient infection by either virus. However, only EcoHIV-infected mice carried HIV products 2LTR DNA and integrated DNA (Fig. 1H), Gag RNA and Tat RNA (Fig. 1I), and *nef* RNA (Fig. 1J), allowing a distinction between EcoHIV and MLV detection. Specific detection of 2LTR and integrated DNA and *tat* and *nef* transcripts provides a quantitative measure of HIV infection and expression *de novo* in mouse brain because these HIV products are absent from the viral inoculum. Finally, to visualize HIV and MLV products in specific cells in mouse brain *in vivo*, brain sections from mice infected with EcoHIV-GFP and MLV-GFP were stained for HIV p24, virus-expressed green fluorescent protein (GFP), and cell-lineage specific markers (Fig. 1K). Consistent with previous observations (27), EcoHIV-infected mice had clearly detectable HIV p24-positive cells in brain parenchyma near perivascular lesions typical of intracerebral HIV infection (Fig. 1K, left panel) and virus-expressed GFP localized to Iba-1-positive macrophages/microglia (Fig. 1K, EcoHIV-GFP panel, merge). In contrast, MLV-GFP localized to irregularly shaped cells near the lateral ventricles, and these cells did not express either astrocytic or monocytoid cell markers (Fig. 1K, MLV-GFP panel, only merged images for either GFP/GFAP or GFP/Iba-1 are shown). Thus, both EcoHIV and MLV replicate well in mouse brain, but EcoHIV infects macrophages/microglia and causes cognitive impairment, and MLV does not.

### EcoHIV, but not MLV, induces microglial activation and inflammatory responses in the basal ganglia region but not apoptosis.

**FIG 2:**
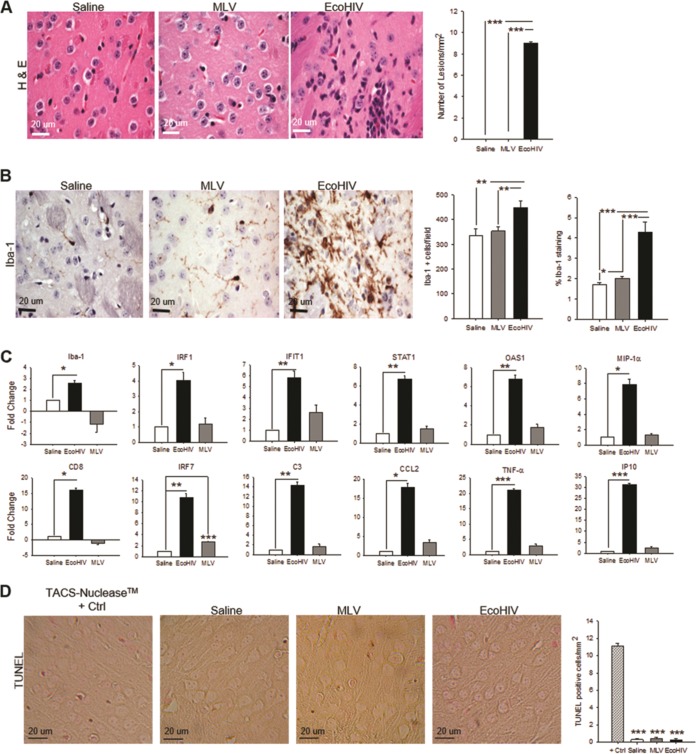
EcoHIV, but not MLV, infection results in pathological changes in the brain and induction of selected host inflammatory and immune activation genes in brain tissue. Mice were inoculated intracranially with EcoHIV, MLV, or saline (three to four mice/group); 2 weeks later, the brains were fixed in paraformaldehyde (PFA), embedded in paraffin, sectioned, and stained or processed for TUNEL assay. The photomicrographs show representative fields from the right basal ganglia region in the vicinity of the injection site in each experimental system. (A) H&E staining depicting inflammatory cell infiltration in EcoHIV-infected mice and no apparent pathology in either MLV or PBS controls. (B) IHC staining for macrophage/microglia marker Iba-1 indicating intense microglial activation in EcoHIV-inoculated mice but minimal activation in MLV infection and saline control. (C) In a separate experiment from that shown in Fig. 1, mice (*n* = 5 per group) were intracranially inoculated with EcoHIV (black bar), MLV (gray bar), or saline (open bar); 2 weeks later, the mice were sacrificed, and total cellular RNAs from brain tissues were compared by RT-QPCR for the expression of genes known to be involved in HIV-1 neuropathogenesis in infected people. The results are shown as the mean fold change ± the SEM versus saline controls set as 1.0. (D) TUNEL staining depicting no significant cell death in any experimental groups. Scale bars are indicated in the bottom left corner of images, and quantifications are shown to the right of each panel (*, *P* < 0.05; **, *P* < 0.01; ***, *P* < 0.001).

We next examined brain tissues from EcoHIV- and MLV-infected mice for evidence of neuroinflammation, which generally accompanies HIV-mediated brain disease (34). The basal ganglia region, the site of viral inoculation, was tested for morphology and microglial activation on paraffin-embedded brain sections, and RNA extracts from this region were tested for cellular inflammatory and interferon-related gene expression (Fig. 2A to C). Brain sections from EcoHIV-infected but not MLV-infected mice showed perivascular lesions defined as infiltrates of mononuclear cells (*P* < 0.0001/saline versus EcoHIV, *P* < 0.0001/MLV versus EcoHIV) and exhibited marked macrophage/microglial cell activation (*P* < 0.0001/saline versus EcoHIV, *P* < 0.0001/MLV versus EcoHIV-percent stained) and increased density of activated cells (*P* = 0.007/saline versus EcoHIV, *P* = 0.006/MLV versus EcoHIV-cells/field) (Fig. 2A and B). Consistent with these results, only brains from EcoHIV- but not MLV-infected mice showed significant upregulation of all 12 inflammation-related genes selected for this study (Fig. 2C). MLV induced only one of the genes tested, *IFN Regulatory Factor-7* (IRF7), confirming the difference in brain pathology profiles between EcoHIV and MLV. The results also exclude the MLV envelope protein in EcoHIV as a potential cause of brain pathologies and NCI seen in EcoHIV infection. Finally, because HIV replication in the brain and neuroinflammation usually correlate with cellular apoptosis (35), we also tested duplicate brain sections from EcoHIV- and MLV-infected mice analyzed in Fig. 2A and B for presence of apoptotic cells in the basal ganglia region by TUNEL (terminal deoxynucleotidyltransferase-mediated dUTP-biotin nick end labeling) assay (Fig. 2D). No cell death was detected in MLV-infected brains (Fig. 2D). Surprisingly, however, brain tissues from EcoHIV-infected mice were also TUNEL assay negative (Fig. 2D), despite significant activation of inflammation related genes in the brain (Fig. 2C). Together, the results show that the intracerebral expression of EcoHIV but not MLV induces cognitive impairment in mice, that the impairment does not involve cell death in the regions tested, and that the difference in cognitive disease outcomes of the two viruses is likely due to expression of HIV-specific gene products and not the envelope gene shared by both viruses.

### EcoHIV replication in mouse brain is required for induction of cognitive impairment.

**FIG 3:**
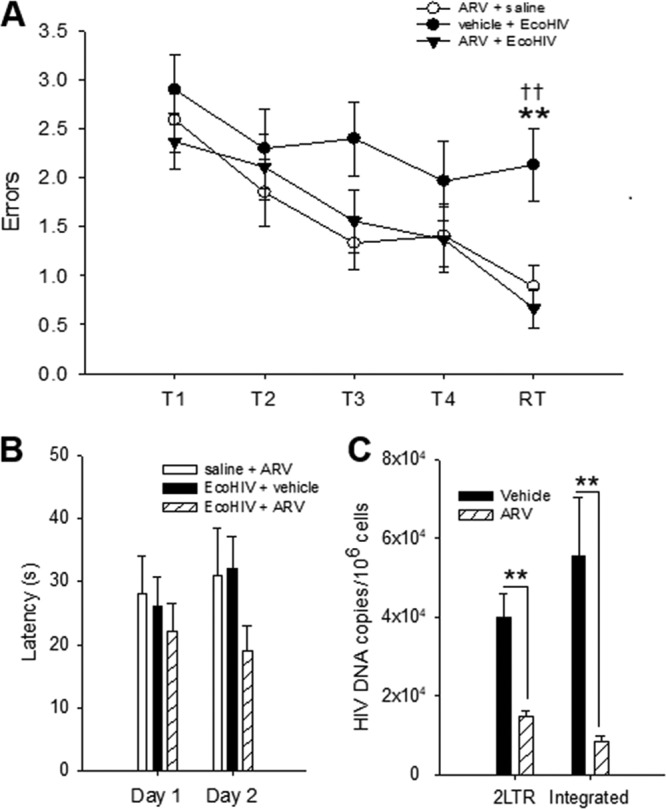
EcoHIV-induced NCI requires viral replication. Mice (*n* = 10 per group) were administered ARV or vehicle by gavage starting 3 days before intracranial inoculation of EcoHIV or saline. All groups were tested in the RAWM test 10 days after infection and subsequently analyzed for virus burdens in brain tissues as described for Fig. 1. (A) Mean errors ± the SEM from the last 3 days of RAWM testing for EcoHIV + vehicle (filled circles), EcoHIV + ARV (filled triangles), and saline + ARV (open circles) systems. T1 to T4 denotes acquisition trials and RT denotes the retention trial performed after a 30 min delay. F_(2,83)_ = 8.118, *P* < 0.001/R; *t*(2) = 3.146; **, *P* < 0.01; EcoHIV + vehicle versus saline, *t*(2) = 3.708; ††, *P* < 0.01; EcoHIV + vehicle versus EcoHIV + ARV. (B) Mean latencies ± the SEM for finding the visible platform on trial 4 on days 1 and 2 of visible platform testing for EcoHIV (filled bar), EcoHIV + ARV (hatched bar), and saline (open bar) infected mice. (C) HIV 2LTR and integrated DNA burdens in brain tissues from EcoHIV-infected + ARV (hatched bar)- or vehicle (filled bar)-treated mice tested by QPCR and shown as mean copies ± the SEM (**, *P* < 0.01).

To determine role of HIV replication in mouse brain in NCI induction, mice were subjected before and during intracerebral EcoHIV infection to daily gavage of a cocktail of three antiretroviral drugs (ARV) used in clinical practice (36). ARV prophylaxis clearly mitigated impairment of memory acquisition during the learning phase and completely prevented impairment in the retention phase (RT) in the RAWM test (significant main effect of trial F_(4,419)_ = 7.440 [*P* < 0.001] and treatment F_(2,419)_ = 8.075 [*P* < 0.001]) (Fig. 3A). ARV treatment alone had no effect on mouse cognition in this test, and all animal groups tested were equally adept in finding the visible platform (Fig. 3A and B). ARV prophylaxis also largely but not completely prevented EcoHIV infection in the brain, as indicated by significant reduction in the HIV 2LTR (*P* = 0.0023) and integrated DNA (*P* = 0.0094) brain burdens compared to untreated mice (Fig. 3C). These results indicate a link between the extent of HIV infection in mouse brain and the induction of NCI in infected animals.

### Murine HIV-NCI persists despite host seroconversion and reduction in virus expression and neuroinflammation.

**FIG 4:**
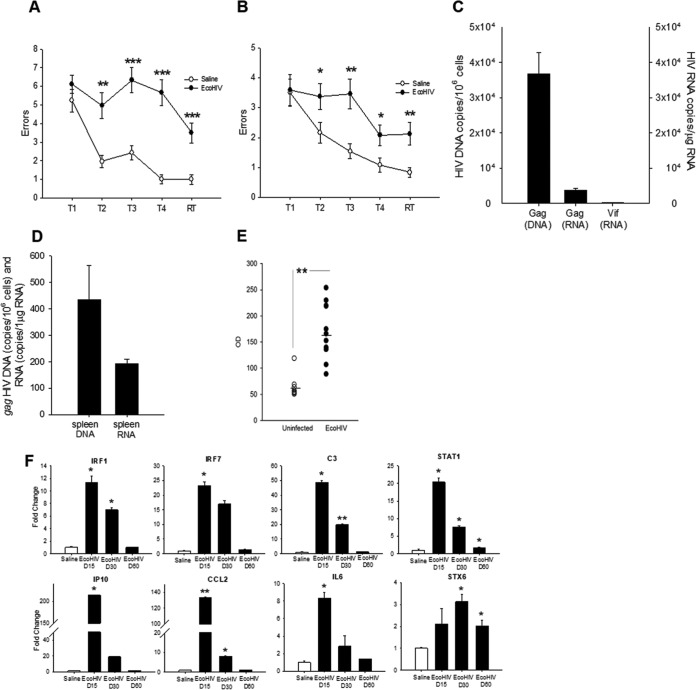
EcoHIV infection in the brain and virus-induced NCI are maintained despite host seroconversion. In two separate experiments, mice (*n* = 10 to 12 mice per group) were intracranially inoculated with EcoHIV or saline. (A and B) Mean numbers of errors ± the SEM made on the last 3 days of RAWM testing for EcoHIV (filled circles) and saline (open circles) inoculated mice 10 days (A) and 2 months (B) postinoculation. T1 to T4 denote acquisition trials, and RT denotes the retention trial performed after a 30-min delay. (C) HIV Gag DNA, Gag, and Vif RNA burdens tested by QPCR/RT-QPCR in brain tissues from EcoHIV-infected mice 2 months after infection, shown as mean copies ± the SEM. (D) HIV Gag DNA and Gag RNA burdens tested by QPCR/RT-QPCR in spleen cells from EcoHIV-infected and control mice 2 months after infection, shown as mean copies ± the SEM. (E) Titers of HIV Gag-specific serum IgG antibodies in mice inoculated intracranially with EcoHIV (closed circles) or saline (open circles) and tested 2 months after infection. Horizontal line indicates mean. **, *P* < 0.01. (F) In a separate experiment, mice (*n* = 3 per group) were intracranially inoculated with EcoHIV; at 15, 30, and 60 days postinfection, mice were sacrificed, and total cellular RNAs from brain tissues were compared by RT-QPCR expression of genes known to be involved in HIV-1 neuropathogenesis in infected people. The results are shown as the mean fold changes ± the SEM versus saline controls set as 1.0.

Mild HIV-NCI is a lifelong condition in infected people with stable control of HIV replication (6). To test the persistence of mouse HIV-NCI, mice were infected by intracerebral injection as described in Fig. 1, and groups were tested for NCI 10 or 60 days after virus inoculation (Fig. 4). As expected, the animals tested 10 days after infection manifested significant impairment of memory acquisition and recall in RAWM (*P* = 0.0013/T2, *P* < 0.0001/T3, *P* < 0.0001/T4, and *P* = 0.0006/RT) (Fig. 4A), reproducing the findings of Fig. 1 and 3. The group of infected mice tested 2 months after infection also showed significant impairment (*P* = 0.0486/T2, *P* = 0.0036/T3, *P* = 0.0306/T4, and *P* = 0.0064/RT), suggesting that HIV causes lasting cognitive damage in mice (Fig. 4B). HIV DNA and RNA burdens in the brain declined between 10 and 60 days after infection, but transcriptionally active HIV was still detectable at the later time point (compare Fig. 4C to Fig. 1). Low levels of HIV DNA and RNA were also detected in the spleen cells of intracerebrally infected animals (Fig. 4D) and anti-HIV Gag antibodies were found in serum 6 weeks after infection (Fig. 4E), indicating that HIV can disseminate from the brain to peripheral tissues and induce adaptive antiviral immune responses. Finally, because induction of murine HIV-NCI by HIV was accompanied by marked induction of genes controlling inflammatory and innate immune responses (Fig. 2), we tested whether these responses persisted as observed for the cognitive disease. Mice were infected intracerebrally, and groups were tested for changes in expression of selected inflammatory and interferon-related genes 15, 30, or 60 days after infection (Fig. 4F). The expression of genes encoding major inflammatory determinants tested including cytokine interleukin-6, complement component C3, and chemokine CCL2 declined from the high levels 15 days after infection to control values 60 days after infection (Fig. 4F). Similar declines were observed for transcripts of several type I interferon-related genes tested, including IRF1, IRF7, and IP10. The expression of the transcription factor STAT1 also declined over time, but it remained significantly elevated above control 2 months after infection. In contrast, the expression of syntaxin-6 (STX6), a regulator of intracellular membrane trafficking (37), increased 30 days after HIV infection and remained above control levels 30 days later (Fig. 4F). These results suggest that HIV infection of immunocompetent mouse brain causes cognitive disease that remains stable despite normalization of expression of multiple inflammatory genes tested in the brain.

### Murine HIV-NCI correlates with hippocampal synaptodendritic injury and neuronal dysfunction but not neuronal apoptosis in the region.

**FIG 5:**
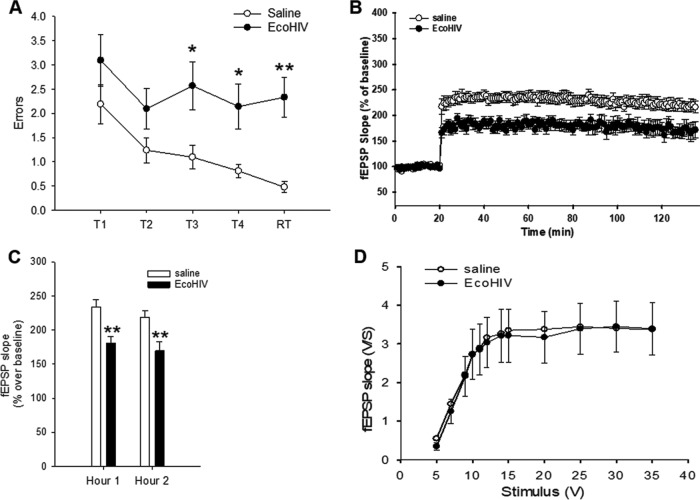
Infected mice with HIV-NCI have reduced LTP in the hippocampus. Mice were inoculated intracranially with EcoHIV or saline (10 animals per group); 10 days later, the animals were tested for NCI in RAWM and then returned to their cages. After a period of rest, pairs of infected and control mice were sacrificed, and hippocampal slices were prepared for electrophysiological measurements. (A) Mean number of errors ± the SEM made on the last 3 days of RAWM testing for EcoHIV (filled circles) and saline (open circles). T1 to T4 denote acquisition trials, and RT denotes the retention trial performed after a 30-min delay. *, *P* < 0.05; **, *P* < 0.01. (B) Summary graph of LTP in saline (open circles) and EcoHIV (filled circles) hippocampal slices. The average fEPSP slope is normalized to the baseline value (ANOVA, *P* = 0.0006). (C) Quantification of the last 10 min of the first hour and the second hour of LTP recordings. **, *P* < 0.01. (D) Summary graph of field input-output relationships for saline (open circles) and EcoHIV (filled circles) hippocampal slices. Both groups showed similar relationships (*P* > 0.05).

We next examined neuronal function and integrity in the hippocampus area, the site involved in visuospatial memory deficits seen in HIV-positive individuals on ART (38, 39) and in peripherally EcoHIV-infected mice (23, 26). For functional studies, intracerebrally EcoHIV-infected mice were first tested for manifestation of learning/memory impairment in RAWM 10 days after infection (Fig. 5A); subsequently, pairs of infected and saline-inoculated controls were serially sacrificed over the following 2 weeks for LTP determination in the CA1 area of acute hippocampal slices (Fig. 5B to D). Consistent with the results shown in Fig. 1, 2, and 4, infected mice suffered significant memory impairment compared to saline controls (*P* = 0.0207/T3, *P* = 0.0171/T4, and *P* = 0.001/RT) (Fig. 5A). The impaired mice also showed significantly reduced LTP in the CA1 area of hippocampal slices compared to controls; both the early and late phases of LTP were affected (*P* = 0.0015/hour 1 and *P* = 0.0086/hour 2) (Fig. 5B and C). In contrast, baseline synaptic transmission and input-output curves were similar in infected and control mice (Fig. 5D), suggesting that HIV brain infection in this model impacts long-term synaptic plasticity and not basal synaptic transmission.

**FIG 6:**
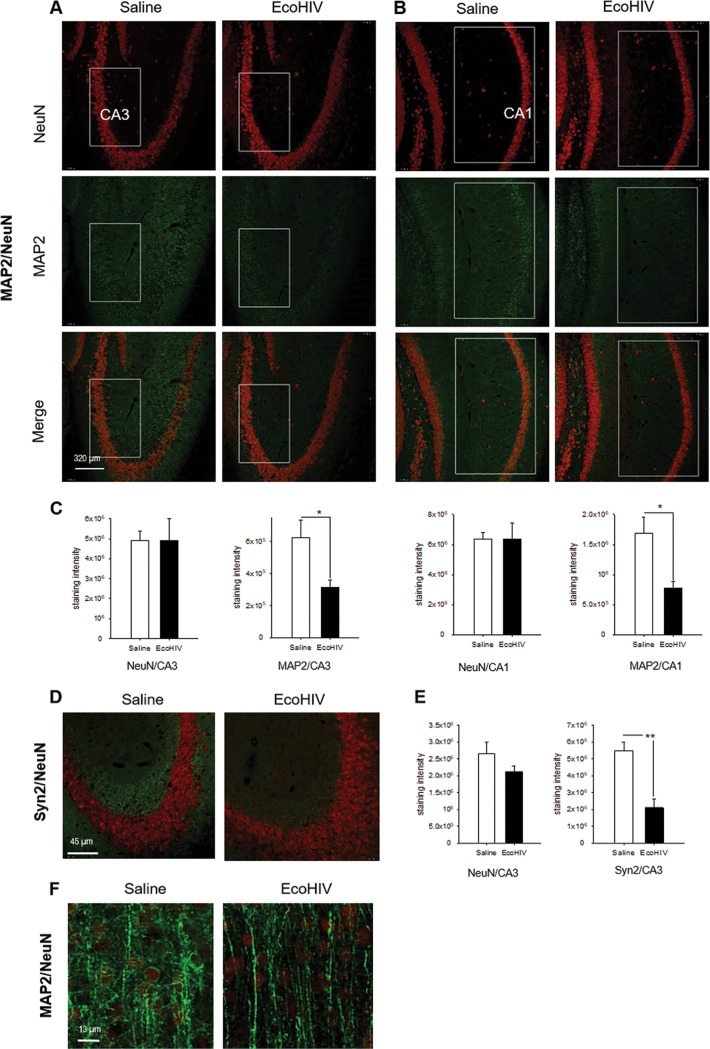
EcoHIV-infected mice show loss of hippocampal dendrites and presynaptic terminals with preservation of neuronal cell bodies. In a separate experiment, mice (*n* = 3 per group) were intracranially inoculated with either saline or EcoHIV; at 14 days postinfection, the mice were sacrificed and perfused with PFA, and frozen brain sections were prepared and double stained with NeuN and MAP2 or Syn2. (A and B) Representative 20× images from the CA3 and CA1 hippocampal regions, respectively, of control (saline) and EcoHIV-infected mice stained for NeuN (top panels), MAP2 (middle panels), and merge (bottom panels). White boxes indicate regions that were quantified, and signal quantification results normalized per image area are shown in panel C. *, *P* < 0.05. (D) Representative merged images from the CA3 region stained with NeuN (red) and Syn2 (green). Original magnification, ×40. (E) Quantification of NeuN and Syn2 fluorescence. (F) Representative merged Z-stack images (each digitally reconstructed from 50 images) showing, at high resolution, MAP2 (green) and NeuN (red) staining of entorhinal cortex dorsal to CA1. Original magnification, ×65.

**FIG 7:**
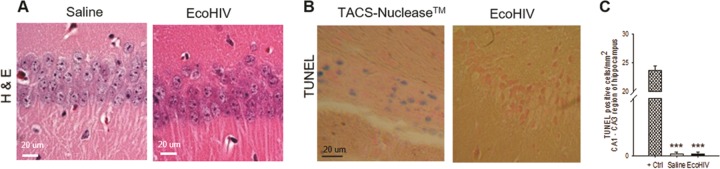
Neuronal loss and apoptosis are not observed during the hippocampal synaptodendritic injury in EcoHIV-infected mice. The brains from three control or EcoHIV-infected mice in the experiment shown in Fig. 6 were fixed in PFA and paraffin embedded, and thin sections from the hippocampus region were either stained with H&E (A) or processed for TUNEL assay (B). Representative images from the same area are shown. Scale bar, 20 μm. (C) Results of TUNEL assay quantification.

In parallel, we examined the extent of physical neuronal injury in the hippocampus 2 weeks after intracerebral infection of mice, the time of initial HIV-NCI manifestation in these animals. Several evaluations were performed. First, we used immunofluorescence staining and confocal microscopy to quantify the expression of the dendritic/axonal marker protein MAP2 (40, 41) and neuronal nuclear protein NeuN in the hippocampi of infected and control animals (Fig. 6A to C). Compared to uninfected mice, EcoHIV-infected animals showed significant decline of about 50% in the intensity of MAP2 staining in hippocampal CA1 and CA3 regions (*P* = 0.0102/CA1 and *P* = 0.0337/CA3), while NeuN detection was unchanged, suggesting dendritic dearborization in the hippocampus. To determine whether reduction in MAP2 staining is accompanied by reduced detection of presynaptic termini, duplicate sections were costained with NeuN and mouse presynaptic marker Syn2 (42), and the fluorescence intensity was quantified (Fig. 6D and E). The Syn2 staining was visibly diminished in EcoHIV-infected mice compared to control mice (saline) throughout the hippocampus, as exemplified in representative merged images (NeuN, red; Syn2, green) from the CA3 area in Fig. 6D. The decline in Syn2 marker detection in infected animals was significant compared to uninfected animals (*P* = 0.0047), while NeuN detection was not significantly changed (Fig. 6E), suggesting presynaptic terminal loss paralleling dendritic loss in the area. Because MAP2 antibody also stains axons, we extended the MAP2/NeuN costaining to the cortical region dorsal to CA1; and for better resolution we conducted Z-stacking (Fig. 6F). As shown in a representative merged image from control mice (saline), pyramidal neurons in this area had clearly defined axons (green), expansive dendritic arbors (green), and visible NeuN-positive (red) neuronal cell bodies (Fig. 6F). In EcoHIV-infected mice, neuronal bodies and axons were still clearly visible, but dendritic arbors were largely diminished, suggesting selective loss of dendrites but not axons. Finally, in an alternative approach to determine the extent of hippocampal neuropathology in infected mice, paraffin-embedded sections from this region were stained with hematoxylin and eosin (H&E), and adjoining sections were tested in a TUNEL assay for cellular apoptosis (Fig. 7). Visual examination of H&E-stained sections suggested normal hippocampal neuronal morphology in infected mice compared to controls (Fig. 7A), while TUNEL assay results showed a minimal number of apoptotic nuclei in infected animals (Fig. 7B and C). Together, the results shown in Fig. 6 and 7 suggest that EcoHIV infection of mice causes a largely nonapoptotic synaptodendritic injury in the hippocampus that correlates with hippocampal neuronal dysfunction and memory impairment.

## DISCUSSION

Our work links HIV expression in immunocompetent mouse brain to memory impairment and neuronal dysfunction in the absence of significant cell loss in the hippocampus, revealing a novel nonneurotoxic function of HIV in cognitive impairment. These findings advance understanding of the HIV role in neuropathogenesis and may inform design of interventions.

Intracerebral infection of conventional mice with EcoHIV allows efficient HIV expression in the brain accompanied by cell activation and inflammatory responses (24, 27). We now show that this infection causes cognitive abnormalities resembling murine NCI in peripherally infected mice (23, 26). Spatial memory dysfunction in the RAWM implies hippocampal injury (30), and a deficient working memory suggests the disruption of executive functions involved in sequential processing of information acquired during learning (29, 43, 44). The fear circuit impairment indicates respective injury to hippocampus and amygdala and possibly a faulty connectivity with prefrontal cortex (PFC) for memory consolidation and recall (32, 33). Together, the cognitive impairments in intracerebrally and intraperitoneally EcoHIV-infected mice resemble, respectively, the HAD-independent cognitive impairments observed initially in AIDS patients (13, 14) and currently (and overwhelmingly) in patients on HIV-suppressive ART (6, 45–47). As in these patients, the cognitive domains affected imply widespread viral injury to neuronal networks spanning the PFC to medial lateral lobe (46). Notably, control mobility tests in RAWM (Fig. 1B) indicated that infected mice lack the gross motor impediments of patients with HIV dementia (48, 49). The dopaminergic neuronal damage in EcoHIV-infected mice (50) could affect fine psychomotor abilities seen in NCI (1), but this possibility has not been tested. Further studies are needed to determine the full range of cognitive and motor abnormalities in this model.

Several observations in the present work link HIV expression in the brain to memory impairment, including prevention of HIV brain infection and NCI with systemic antiretroviral prophylaxis; the persistence of brain infection along with cognitive disease, reminiscent of observations in HIV-suppressed NCI patients (20, 51); and the real-time correlation between NCI and expression of *tat* and *nef* transcripts that encode proteins implicated in HIV neuropathogenesis (52). While we did not attempt to detect Tat protein in the brain here, its production in EcoHIV-infected mice was reported previously (22). Notably, the intracerebrally inoculated MLV used as a control virus replicated well in mouse brain but did not cause NCI, confirming that HIV-specific functions were required for NCI induction in this model. Because MLV and EcoHIV share the gp80 envelope gene (22), this control also demonstrates that murine NCI cannot be attributed to MLV envelope in EcoHIV. The detection of HIV proteins in brain monocytic cells here and previously (27) suggests that these cells are the main targets for EcoHIV expression in this model, demonstrating commonality of murine NCI and HAND through the proposed central role of monocytic cells for HIV infection in the brain and neuropathogenesis (23, 53, 54). Conversely, the failure of MLV to induce NCI here could be attributed to its inability to infect and activate brain myeloid cells. A difference in EcoHIV and MLV tropism to monocytic cells was observed previously and found not to solely depend on the cellular display of CAT-1, the receptor for MLV and EcoHIV (23). Murine retrovirus tropism is conferred by both viral envelope and LTR (55), suggesting that LTR activity and host cell transcription factors contribute to EcoHIV tropism in mice. Finally, it should be noted that the spleen was not infected in intracerebrally inoculated mice when NCI was first detected, suggesting that infection confined to the brain was sufficient for initiating the cognitive disease development. Low levels of HIV were detected in the spleen 2 months after infection (Fig. 4), suggesting dissemination of virus from the brain and likely the source of antigen for the observed seroconversion of intracerebrally inoculated mice. This virus could also contribute to NCI, for example, by promoting chronic systemic inflammation (23, 56–58).

Considering that we intentionally maximized EcoHIV infection in mouse brain, observing both neuroinflammation and expression of HIV genes encoding HIV neurotoxins, it was surprising that we saw minimal neuronal loss in the basal ganglia region, the site of virus inoculation, and in hippocampus and entorhinal cortex, the sites required for the cognitive functions tested here. This is a different neuropathological outcome than in HIV and SIV encephalitis, where neuronal death prevails and is thought to result from combined effects of viral neurotoxins and neuroinflammation (28, 59–61). A recent study reported loss of dopaminergic but not nondopaminergic neurons in the substantia nigra and subventricle brains of EcoHIV-infected mice (50), suggesting that EcoHIV infection may reproduce some of the neuron type-specific neurotoxic functions of HIV (18). However, our results clearly associate HIV brain infection and spatial memory impairment in real-time with nonnecrotic hippocampal neuronal injury reflected in extensive dendritic dearborization and reduced synaptic density. LTP measurements in hippocampal slices *ex vivo* link this injury to hippocampal synaptic dysfunction. Synaptodendritic simplification was also observed in entorhinal cortex, suggesting diffuse nature of HIV-mediated neuronal injury and potential functional disruption of the major neuronal pathway for spatial navigation connecting between thalamus and PFC (62). Together, these results suggest that losses of synapses, dendritic integrity, and neuronal function in the hippocampus and not neuronal death are the primary neurobiological defects responsible for memory deficits in this model. We recently reached similar conclusions in studies in peripherally EcoHIV-infected mice by showing that memory impairment in these mice can be reversed with appropriate interventions (25, 26). In particular, the observation that intranasal insulin treatment rapidly restores hippocampal dendritic arbors and functional memory clearly indicates that neuronal cells required for spatial memory recording *s*urvive HIV insult in this model and can regain full functionality (26). Our conclusions are concordant with the view of mild cognitive impairment as “mainly a synaptodendritic disorder” that is largely independent of neuronal loss (18). Notably, this insight originated from analysis of brain tissues in AIDS patients with active HIV expression and neuroinflammation (13, 18), akin to the system described here. The extent of neurodegeneration in untreated HIV-positive patients was recently shown to correlate directly with plasma HIV burdens and inversely with CD4^+^ T cell levels (63). HIV-positive individuals on ART have normal CD4 levels and minimal neurodegeneration, regardless of whether they manifest NCI (15, 63, 64). However, their cognitive impairment still correlates best with a combined reduced display of dendritic and synaptic markers (19), similar to the findings here. To our knowledge, EcoHIV-infected mice with memory impairment are the only animal model of HIV infection that reproduces, at least regionally in the brain, the nonnecrotic structural-functional injury of neurons that may underlie NCI in individuals on ART.

Animal models can only approximate the processes involved in human disease (65). Further studies are needed to determine the basis of neuronal injury in the PFC and other anatomical sites involved in cognitive deficits in the EcoHIV model. However, our recent reports (23, 25, 26) and this one allow us to make two general points on HIV neuropathogenesis in the ART era. First, HIV expression in brain tissue is sufficient, and may be the only trigger required, for the induction of cognitive impairment. Because HIV enters the human brain early in infection (66, 67), and cognitive disease can develop even when ART is initiated within weeks of infection (68), new treatment paradigms will have to be developed to prevent the disease. Second, our data reveal a nonneurotoxic function of HIV in cognitive impairment distinct from HIV neurotoxicity in dementia. We propose that the ability to cause this injury is intrinsic to multifunctional HIV proteins such as Tat or Nef (52, 69), as exemplified by nonneurotoxic synaptic dysfunction and memory impairment in young inducible-Tat transgenic mice (70). Nonnecrotic synaptodendritic damage is likely superseded during the extensive neuroinflammation in HIVE by frank cell death caused by cellular neurotoxins and brain homeostasis breakdown, as well as injurious viral proteins (48, 53, 71). However, when virus control in the brain is provided by immune responses (27, 72) or effective ART, noncytopathic neuronal injury may prevail, at least temporarily, as indicated by stable NCI course in the majority of patients followed in longitudinal studies for 3 to 4 years (3, 9, 10). That neurons can survive HIV insult under some host conditions has important implications for control of the disease. If neurons are injured but viable, reversal of synaptodendritic injury and neuronal functionality is possible as shown after moderate traumatic brain injury in mice (73), insulin-like growth factor-2 treatment in APP transgenic mice (74), and intranasal insulin treatment of EcoHIV-infected mice (26). Since an HIV cure is still remote, mitigating NCI may be the only option to prevent deterioration of the disease with aging (3, 10).

## MATERIALS AND METHODS

### Mice.

Male, 8-week-old 129X1/SvJ mice (Jackson Laboratory, Bar Harbor, ME) maintained under standard mouse husbandry conditions were used exclusively. Discomfort, distress, and injury to the animals were minimized. Animal studies were approved by the Icahn School of Medicine at Mount Sinai Institutional Animal Care and Use Committee (IACUC) in full compliance with the U.S. Animal Welfare Act and Public Health Service (PHS) policies and under Institutional PHS animal welfare assurance D16-00069.

### Infectious virus stocks.

The viruses used were EcoHIV/NDK (22), EcoHIV/NL4-3-GFP (27), and ecotropic MLV (75), referred to in the text as EcoHIV, EcoHIV-GFP, and MLV, respectively, as well as GFP-expressing MLV (MLV-GFP), kindly provided by Y. Sabo and S. Goff, Columbia University. Virus stocks were prepared, and titers were determined as described previously (23). MLV stocks were confirmed for infectivity in rat XC cells (76) and normalized for infection to EcoHIV stocks by shared envelope RNA content (23, 77).

### Infection of mice, antiretroviral drug treatment, and tissue collection.

Mice were infected by stereotaxic intracranial injection into the right hemisphere basal ganglia as previously described (24, 27) using 10^6^ pg of p24 of EcoHIV or virus RNA equivalents of MLV in 10 μl; the injection rate was 0.5 μl/min, and saline served as a control. Behavioral testing began 10 days postinfection. Antiretroviral (ARV) drugs were administered by gavage as a mixture of 100 mg/kg/day each of abacavir, indinavir, and raltegravir in water, starting 72 h before infection and twice daily until behavioral testing and then daily at 50 mg/kg/day until the end of behavioral testing. The gavage mixture was prepared from clinically available medications as described previously (23). Upon euthanasia, blood, spleens, and brains were removed and prepared for measurement of HIV burden, cellular gene expression, and microscopy as described previously (26, 27).

### Mouse behavioral tests.

Learning and memory were evaluated in groups of 8 to 10 mice using the radial arm water maze (RAWM) and auditory-cued fear conditioning (FC) tests as described previously (23, 26, 29). Briefly, RAWM testing consisted of four training trials (T), followed by a retention trial (RT) administered after a 30-min rest, repeated daily until control mice reached asymptotic performance of one error or fewer on trials T4 and RT. Errors for the last 3 days of testing were averaged for statistical analysis. The hidden platform tests were followed by measuring the latency for finding a visible platform as a control for possible effects of treatment on animal vision, motivation, and motor ability. FC testing was conducted using an SDI Freeze Monitor (San Diego Instruments, San Diego, CA). Conditioning sessions included three consecutive pairings of 10-Hz sound signals and 0.7-mA electric shock signals; cued associative fear memory was measured the following day in a novel context by presenting sound signal alone. Results are shown as the mean total percentage of time spent freezing pre- and postcue on both the conditioning and the cued memory days.

### Measurement of virus burdens and cellular gene expression.

The procedures for harvesting mouse tissues, preparation of cellular DNA and RNA, and detection of EcoHIV/NDK *gag* transcript and 2LTR and *gag* DNA by real-time quantitative PCR (QPCR) were described previously (23, 78). QPCR detection of gp80 *env* RNA transcript and DNA common to EcoHIV and MLV by QPCR were as described previously (77), and detection of spliced *nef* transcripts by conventional RT-PCR and Southern blotting were as described previously (27). The spliced *tat* transcript was detected by RT-QPCR (79) with the following modifications: the reaction mixture contained custom-designed forward primer ND-tat-F (5′-CCT AGG ACT GCT TGT AAT AAG TGT-3′), reverse primer ND-tat-R2 (5′-GTC GGG TCC CCT CGG GAC TGG GAG-3′), and probe ND-tat-P [5′-(FAM)-AAA GGC TTA GGC ATC TC-(MGBNFQ)-3′]. A standard curve for quantitation of *tat* copy number was constructed using graded numbers of plasmid TA-NDK-*tat* containing a spliced NDK *tat* cDNA fragment amplified with ND-tat-F and ND-tat-R2 primers and cloned into the TA-cloning pCR 2.1 vector (Thermo Fisher Scientific, Waltham, MA). Integrated EcoHIV/NDK proviral DNA was detected by a nested QPCR, as described previously (23). Samples for QPCR were run in duplicate in the AB7300 real-time thermal cycler (Thermo Fisher Scientific). RNA and DNA QPCR reactions were normalized by amplification of glyceraldehyde 3-phosphate dehydrogenase (GAPDH) using a kit from ABI. Data were analyzed using the 7300 System software according to the manufacturer’s instructions. Changes in the expression of genes from mouse brain samples were analyzed by QPCR using TaqMan chemistry and probes from the Universal Probe Library (Roche, Indianapolis, IN), as previously described (27, 80). Relative quantification employed the comparative threshold cycle method (Applied Biosystems, technical bulletin 2).

### Histopathology, immunohistochemistry, and ELISA.

Mouse brains were fixed in 4% paraformaldehyde, dehydrated, embedded in paraffin, sectioned in 6-μm coronal sections, and either stained with H&E for histopathological assessment or used for the detection of HIV p24 and selected brain cell markers by immunohistochemistry, as described previously (27). The antibodies used were a rabbit polyclonal antibody to glial fibrillary acidic protein (GFAP) from Dako for labeling astrocytes, a rabbit polyclonal antibody to ionized calcium binding adaptor molecule-1 (Iba-1) from Wako (Osaka, Japan) for labeling macrophages/microglia, and a mouse monoclonal antibody 183-H12-5C (NIH AIDS Reagent Program, Germantown, MD; catalog no. 3537) for the detection of HIV-1 p24 antigen. Photomicrographs were taken on a Zeiss Photomicroscope III equipped with a Nikon DN100 digital camera. Quantification of immunostaining was performed using Volocity 5.5 image analysis software (Perkin-Elmer) on 10 to 20 images from multiple brain sections (five to eight images/section from two to three mice/group) and calculating the positive-stain area as a percentage of the total stained area per microscope field for each protein or counting stained cells or lesions per field normalized by image area. Two months after vehicle or EcoHIV injection, serum anti-HIV/NDK IgG was measured by an enzyme-linked immunosorbent assay (ELISA), as described previously (81).

### Immunofluorescence staining and confocal microscopy.

Mouse perfusion, preparation of frozen brain sections, immunofluorescence staining of sections, and confocal microscopy were performed as previously described (26, 27). The antibodies used were chicken anti-GFP (1:500; Thermo Fisher Scientific) for the detection of GFP in EcoHIV/NL4-3-GFP and MLV-GFP, rabbit anti-Iba1 (1:400; Wako) to identify macrophages/microglia, rabbit anti-GFAP antibody (1:500; Thermo Fisher Scientific) to identify astrocytes, rabbit anti-microtubule-associated protein 2 (MAP2) for detection of dendrites (1:150; EMD Millipore, Mahopac, NY), rabbit anti-mouse synapsin II (Syn2) for detection of synapses (1:500; Abcam, Cambridge, MA), and mouse monoclonal anti-neuronal nuclear antigen (NeuN) for the detection of neuronal nuclei (1:150; EMD Millipore), followed by matching Alexa-conjugated secondary antibodies (1:100; Thermo Fisher Scientific). Images including Z-stacking were captured with a motorized Leica TCS SP5 confocal microscope and analyzed using Improvision Volocity software (Perkin-Elmer) as described previously (27). Quantification of images was performed using 10 to 20 images (two to four sections/mouse, three mice/group) measuring the intensity of staining for each protein in the selected areas indicated in Fig. 6C, as described previously (27).

### TUNEL assay for cellular apoptosis in mouse brain.

Cell death was determined on 6-μm paraffin-embedded brain sections using the TACS XL Blue Label kit (Trevigen, Gaithersburg, MD), following the manufacturer’s protocol. For positive control, samples were treated with TACS-nuclease to generate DNA breaks in cells. Slides were examined under a Nikon light microscope and images were acquired by a Leica DFC425 camera. Image analysis was conducted by the LAS V4.0 program (Leica Microsystems, Inc., Buffalo Grove, IL) by counting the positively stained cells per mm^2^ from 10 different fields from each of the three slide replicates for a total of 30 fields per group.

### Electrophysiology measurements in mouse hippocampus slices.

Extracellular field potential recordings were conducted using transverse hippocampal slices (400 μm) essentially as described previously (82, 83). Field extracellular postsynaptic potentials (fEPSPs) were recorded using a stimulating electrode at the Schaffer collateral fibers and a recording electrode at the CA1 stratum radiatum. Basal synaptic transmission was assessed by plotting stimulus voltages (V) against the corresponding fEPSP slopes. Stimulus intensity was set so that baseline responses were approximately 1/3 of the maximum evoked response. Long-term potentiation (LTP) was induced using theta-burst stimulation (four pulses at 100 Hz, with the bursts repeated at 5 Hz and each tetanus including three 10-burst trains separated by 15 s). Responses were measured as fEPSP slopes expressed as percentages of the baseline average.

### Statistical analysis.

Differences in HIV-1 burdens and other parameters between controls, infected, or treated mice were tested by unpaired Student *t* test with *P* values shown by asterisks in figures. RAWM data involving multiple comparisons were analyzed using a two-way analysis of variance (ANOVA) with a Holm-Sidak *post hoc* pairwise comparison. Individual trial data were analyzed using a one-way ANOVA with a Holm-Sidak *post hoc* pairwise comparison. Changes in cellular gene expression in the brain tissues of infected mice were first normalized to respective uninfected controls, with comparisons being made among each group. *P* value representations are indicated (*, *P* < 0.05; **, *P* < 0.01; ***, *P* < 0.001).
